# A comprehensive study of raw and roasted macadamia nuts: Lipid profile, physicochemical, nutritional, and sensory properties

**DOI:** 10.1002/fsn3.2143

**Published:** 2021-01-27

**Authors:** Xing‐Hao Tu, Bang‐fu Wu, Ya Xie, Shu‐Ling Xu, Zong‐Yuan Wu, Xin Lv, Fang Wei, Li‐Qing Du, Hong Chen

**Affiliations:** ^1^ Oil Crops Research Institute of the Chinese Academy of Agricultural Sciences/Key Laboratory of Oilseeds Processing of Ministry of Agriculture and Hubei Key Laboratory of Lipid Chemistry and Nutrition Wuhan China; ^2^ South Subtropical Crop Research Institute Chinese Academy of Tropical Agricultural Science/Key Laboratory of Tropical Fruit Biology Ministry of Agriculture Zhanjiang China

**Keywords:** lipid composition, macadamia nut, physicochemical properties, roasting

## Abstract

Macadamia nuts have high nutritional value and positive health attributes. Changes to the composition and availability of these compounds during roasting contribute to product quality. In this study, changes to the chemical composition of lipids (fatty acids, triglycerides, and free fatty acids) and other phytochemicals were analyzed, and a sensory evaluation was carried out of two major varieties of macadamia nuts planted in China, after roasting. Only small changes in fatty acid (FA) content and a slight decrease in total triglycerides (TAGs) were observed after roasting. The free fatty acid (FFA) content and the peroxide value were increased by roasting. The total available polyphenol content increased by 25.6% and the oxidative stability index of kernels increased by 21.6%. The sensory scores for taste and aroma were doubled by roasting. Overall, the sensory, nutritional quality, and oxidative stability of roasted macadamia nuts were greatly improved, compared with raw nuts.

## Graphical abstract

This research provided a deep insight into the quality control of macadamia nuts during roasting and would help set guidelines for selecting the suitable roasting conditions for macadamia nuts.

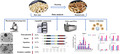

## 1 INTRODUCTION

Tree nuts are consumed as healthy snacks worldwide, either in raw (natural) form or in roasted. They are highly desirable because of their high content of essential amino acids and polyunsaturated fatty acids (Bailey & Stein, 2020; Hu et al., 2019; Xie et al., 2019). Macadamia (*Macadamia tetraphylla*) nuts have a subtle, butter‐like flavor, with a creamy texture (Moreno‐Perez et al., 2011) and they grow on the macadamia tree (*Macadamia* sp.; family *Proteaceae*), which is native to the rainforests of eastern Australia (Wang et al., 2014). In recent years, the macadamia nut industry in China has developed rapidly, with the planting area expected to be 15,000 ha; the trees are widely planted in southern China, especially in Yunnan and Guangxi provinces (Hong et al., 2018; Zhong et al., 2020).

Roasting is the most commonly used processing method for preserving quality and improving the shelf life of macadamia nuts (Buthelezi et al., 2019). This thermal process can improve the flavor, aroma, color, texture, and appearance of macadamia nuts through Maillard browning reactions, or lipid oxidation (Srichamnong & Srzednicki, 2015; Walton & Wallace, 2011). Therefore, roasted kernels with their crisp texture and unique flavor are preferred over raw kernels by consumers. Lipids are the major component of macadamia nuts, accounting for about three‐quarters of the kernel weight, with triglycerides (TAGs) accounting for more than 95% of the lipids (Moreno‐Perez et al., 2011). However, excessive lipid oxidation results in rancidity and deterioration of macadamia nuts, which makes them both unhealthy and undesirable to the consumer (Colzato et al., 2011). Roasting of nuts must be carefully controlled, to avoid excessive lipid oxidation (Chandrasekara & Shahidi, 2011).

Roasting may alter the acid value (AV) and peroxide value (POV) of macadamia nuts, which are indicators of nut rancidity (Bolling et al., 2011). POV and AV are evidence of autoxidation and hydrolytic rancidity, respectively. Hydroperoxides produced by autoxidation react with other nut components, resulting in rancidity (Le Lagadec, 2009). Triglyceride hydrolysis by esterases and lipases produces free fatty acids (FFA) and causes hydrolytic rancidity (Wall, 2010). Acceptable AV values vary for different nuts; for example, AVs of less than 0.5% and 1.0% are acceptable for macadamia nuts and almonds, respectively (Bolling et al., 2011). Unsaturated triglycerides are the most susceptible to enzymic hydrolysis, because their multiple double bonds lower the melting point of the fat and liquid oils are more easily hydrolyzed by lipases than solid fats (Arroyo‐Caro et al., 2016; Wall, 2010). FFA is more susceptible to oxidation than triglycerides (TAGs), resulting in undesirable volatile compounds and off‐flavors, which reduces the quality of macadamia nuts (Toth, 2017).

Macadamia nuts are predominantly consumed roasted. The roasting process is highly likely to induce chemical changes in the lipids and consequential flavor modification. There have been many studies on the effects of roasting on the main nutrients and sensory properties (Bailey & Stein, 2020; Hu et al., 2019). However, there is limited information on the effects on TAGs and FFAs in macadamia nuts. Shotgun lipidomics is a rapid, quantitative technique for analyzing lipid molecular species (Chen et al., 2017; Xie et al., 2019) and has been applied in this study to compare the nutritional quality and lipid oxidation between raw and roasted macadamia nuts. Nuts also contain significant amounts of polyphenolic compounds, which have many beneficial health functions, including high antioxidant capacity, and roasted nuts contain Maillard reaction products, which contribute to flavor, color, and antioxidant capacity (Lin et al., 2016). In this study, changes in physicochemical properties, lipids (fatty acids, triglycerides, and free fatty acids), nonlipid compounds (sterols, polyphenols, etc.), and sensory quality indicators were examined in the two main varieties of macadamia nuts planted in China, both before and after roasting. Our results provided a deep insight into the quality control of macadamia nuts during roasting and would help set guidelines for selecting the suitable roasting conditions for macadamia nuts, to obtain the best quality.

## 2 MATERIALS AND METHODS

### 2.1 Chemicals and reagents

HPLC‐grade hexane, acetonitrile (ACN), 2‐propanol, chloroform (CHCl_3_), methanol (MeOH), ammonium hydroxide (>25%), formic acid (>99%), acetic acid (>99%), heptadecanoic acid, and ammonium acetate were from CNW (Düsseldorf, Germany). Triethylamine (TEA) and N,N‐diethyl‐1,2‐ethanediamine (DEEA) were from Shanghai Aladdin Reagent Co., Ltd. 2‐Chloro‐1‐methylpyridinium iodide (CMPI) was from Sinopharm Chemical Reagent Co., Ltd. 1,2,3‐Tri‐(heptadecenoyl)‐glycerol (TAG‐17:1/17:1:/17:1) was from Avanti Polar Lipids, Inc. (Alabama, USA). Other standards were from Sigma‐Aldrich (St. Louis, MO, USA), unless otherwise stated. Other reagents were analytical grade from Sinopharm Chemical Reagent Co., Ltd. (Shanghai, China). Ultra‐pure water (Milli‐Q, Millipore, Bedford, MA, USA) was used for all experiments.

### 2.2 Preparation of dried raw and roasted materials

Two major varieties of macadamia nuts in China, Guire 1 and Own Choice (O.C), were obtained from the National Field Genebank for Tropical Fruits, South Subtropical Crops Research Institute, Chinese Academy of Tropical Agricultural Sciences. The nuts were dehusked within 24 hr of harvest. Nuts, in the shell, were then stored in polyethylene bags. Fresh nuts (15% dry basis moisture content, db) were stored at −18°C until needed. The nuts were taken out and equilibrated to room temperature for 3–4 hr and then dried in an mechanical convection oven (SHT, Sanxiong Machinery Manufacture Co. Ltd., Shangyu, China) using a regime (hot air‐drying at 35℃ for 2 days, 45℃ for 2 days, 55℃ for 2 days), as described previously (Walton et al., 2013). The moisture content of the nuts was monitored, and the nuts were removed from the oven, when the moisture content had reached 1.5% (db) and designated as raw.

Dried raw nuts were roasted at 125°C for 15 min using an electric rotary oven (X2R, Guangdong Galanz Group Co., Ltd., China), which reduced the moisture content to 1.0% (db), and these nuts were designated as roasted. These roasting conditions were selected based on the nut industry best practice, to avoid quality deterioration (Buthelezi et al., 2019).

### 2.3 Microstructure analysis

For analysis of microstructure, the nut samples were examined by transmission electron microscopy (TEM, H‐7650, Hitachi, Tokyo, Japan), as described previously (Niu et al., 2015). Briefly, the nut samples were fixed, washed and dehydrated, embedded in acetone and Spurr resin. Sections 70–90 nm thick were cut from the embedded tissue using a microtome, double‐stained with uranyl acetate and lead citrate, and then examined.

### 2.4 Physicochemical property analysis

Acid value (AV) was determined by AOCS official method Cd 3a‐63 (AOCS, 2004). Peroxide value (PV) was determined by the AOAC 965.33 method (AOAC, 2005). Water activity (a_w_) was measured with an Aqualab^®^ series 3 TE water activity meter (Decagon Devices Inc., Pullman, WA., U.S.A.).

The oxidative stability index (OSI) was determined in terms of the induction period (IP) with an 892 Professional Rancimat (Metrohm, Switzerland). The nut sample (0.5 g) was heated to 120°C in a reaction tube and subjected to air inflow of 20 L/h. IP was automatically calculated by the instrument's software. Three readings per sample were taken and averaged. Moisture content (AOAC method 925.40), total fat (AOAC method 948.22), ash (AOAC method 950.49), total dietary fiber (AOAC method 985.29), and crude protein (AOAC method 950.48) (AOAC, 2005) were also determined. The total carbohydrate content was calculated by subtracting the sum of the crude protein, total fat, water, and ash from the total weight of the nut flour (Birch et al., 2010).

### 2.5 Lipid concomitants analysis

Ground macadamia nut kernels (3 g) were extracted with hexane/isopropanol (9 ml, 3:2 v/v) under vigorous stirring for 15 min and then filtered under vacuum. The filter residue was re‐extracted twice with hexane/isopropanol (3:2) and then dried with anhydrous sodium sulfate. The filtrate was centrifuged at 8,983 *g* for 10 min. The extracts were dried under N_2_ to yield an oil. The sterol, tocopherol, tocotrienols, thiamine, and squalene contents were analyzed as described previously (Wall, 2010) by HPLC.

The total phenolic content was determined spectrophotometrically using Folin–Ciocalteu reagent at 750 nm, with gallic acid monohydrate as a standard and expressed as mg gallic acid equivalents (GAE) kg^‐1^ of dry weight, as described previously (Buthelezi et al., 2019).

### 2.6 Analysis of fatty acid composition

Macadamia nuts were cold‐pressed (CA59G press, IBG Monforts, Nuremberg, Germany) at room temperature. After centrifugation at 8,983 *g* for 20 min, the oil was transferred to a brown glass container, kept at 4℃, and analyzed within 1 week. A standard procedure with KOH/methanol solution (0.4 mol/L) was used to prepare fatty acid methyl esters (FAMEs) from the oil (Xie et al., 2019; Xu et al., 2018). The FAMEs were analyzed by gas chromatography (Agilent Technologies 7890A, Santa Clara, CA) coupled with a capillary column (HP‐FFAP, 30 m × 0.25 mm × 0.25 μm, Agilent) and a flame ionization detector (FID). Sample (1.0 μl) was injected in splitless mode. The carrier gas was nitrogen with an inlet pressure of 1.7 × 10^5^ Pa. The oven temperature was maintained at 210°C for 1.0 min, then ramped to 230°C at 10°C/min, and then held for 7 min. The temperatures of the injection port and detector were maintained at 250 and 280°C, respectively. The relative content (% w/w) of each FA was calculated (peak area of each fatty acid/peak area of total fatty acids). Heptadecanoic acid (17:0, 70 μl, 5.15 mg/ml) was used as the internal standard to quantify individual fatty acids.

### 2.7 Shotgun‐NL‐ESI‐MS/MS for the analysis of TAGs

Triacylglycerols (TAGs) were determined as described previously (Xie et al., 2019). Briefly, an aliquot (25 mg) of each nut oil sample was dissolved in n‐hexane and diluted to 10 mg/ml. An aliquot (20 μl) of hexane solution was transferred to a glass culture tube, to which the internal TAG standard (17:1/17:1/17:1, 0.1 mg/ml in methanol, 10 μl) was added. Methanol (450 μl), chloroform (450 μl), and NH_4_OH (10%, 100 Μl) were added sequentially and then vortexed for 1 min. The TAG analysis was performed on a shotgun‐ESI‐MS/MS system consisting of a 4,000 Q‐Trap mass spectrometer (AB Sciex, Toronto, Canada) with an ESI source in positive mode.

The TAGs were detected as ammonium ions, [M + NH_4_]^+^, by a series of neutral loss (NL) scans. The scans targeted losses of fatty acyl chains as neutral fragments with NH_3_, including NL285.3 (17:1), NL245.3 (14:0), NL273.3 (16:0), NL271.3 (16:1), NL297.3 (18:2), NL299.3 (18:1), NL301.3 (18:0), NL327.3 (20:1), NL329.3 (20:0), and NL357.3 (22:0), NL385.3 (24:0). The collision gas was nitrogen and the collision energy +25 eV. The collision gas pressure was set on “low.” The declustering potential was +100 V, the entrance potential +14 V, and the exit potential +14 V. The source temperature (heated nebulizer) was 100°C. The curtain gas was set at 20 psi; and the two ion source gases were set at 45 psi. The electrospray capillary voltage was +5.5 kV. The mass spectrum was scanned in the range m/z 500–1200.

### 2.8 Chemical derivatization coupled with NLS‐ESI‐MS/MS for the analysis of FFAs

Determination of FFAs in oil samples was achieved using DEEA derivatization and analysis by shotgun‐ESI‐MS/MS (as above). Derivatives of FFAs were detected using the NL method (NLS 73 Da) in positive‐ion mode, as described previously (Liu et al., 2018). Briefly, an aliquot (10 mg) of each oil sample was dissolved in n‐hexane and diluted to 0.1 mg/ml. An aliquot (20 μl) of diluted oil sample, internal standard (heptadecenoic acid, C17:1, 1 μmol/L in CAN, 10 μL), ACN (850 μl), TEA (20 μmol/ml, 30 μl), and CMPI (20 μmol/ml, 20 μl) was added sequentially and vortexed for 1 min. Finally, DEEA (20 μmol/ml, 50 μl) was added to label the FFAs. The derivatizing reaction was performed in an ultrasonic bath (Ultrasonic Cleaner, Jeken Ultrasonic Cleaner Limited, China), for 5 min at 40°C. The ultrasound frequency and power were 40 kHz and 500 W, respectively. The resultant mixture was dried under nitrogen gas, and then, CHCl_3_ (1 ml) and formic acid–water (2 ml, 10:90 v/v) were added. After vortexing the sample for 2 min, the upper phase was removed and the extraction step repeated three times. Finally, the chloroform phase was dried under a stream of nitrogen and redissolved by ACN (1 ml). The collision energy (CE) and declustering potential (DP) were set at +32 V and +75 V. Collision energy spread and collision cell exit potential were all set at 10 V. The curtain gas pressure was 20 psi, and the two ion source gases were set at 40 psi. The mass spectrum scanning was operated in the range of m/z 200–500.

### 2.9 Sensory evaluation

Sensory evaluation was carried out as described previously (Buthelezi et al., 2019). The panelists (10 males and 10 females) ranging from the age of 30 to 50 were requested to evaluate the sensory attributes of “Guire 1” and “O.C” kernels, including their texture, aroma, rancid taste, and overall appearance. The quality of kernels was rated using the hedonic scale; the specific evaluation criteria are shown in Table 1. After scoring, the average value of each index is used for analysis of variance by SPSS 21.0 software (SPSS Inc., Chicago, IL).

**TABLE 1:** The hedonic scale of the quality of kernels

Score	1	2	3	4	5
Texture	Very smooth	Smooth	Neutral	Crispy	Very crispy
Aroma	Poor	Fair	Good	Very good	Excellent
Overall appearance	Poor	Fair	Good	Very good	Excellent
The rancid taste	None	Slight	Moderate	Moderately severe	Severe

### 2.10 Data and statistical analysis

The mass data acquisitions were performed using Analyst 1.5 software (AB Sciex, USA) and Lipidview software ((AB Sciex, USA) for peak extraction and analysis of the acquired data. All experiments were performed in triplicate. The statistical analysis was performed by using SPSS 21.0 (SPSS Inc., USA). One‐way analysis of variance (ANOVA) with Duncan's multiple range test was used for multiple comparisons, and differences between two groups were evaluated using the Student's t tests. Differences among groups were considered statistically significant at *p* < 0.05. All the figures with means and standard deviations for all values were computed in GraphPad prism 6.

## 3 RESULTS

### 3.1 Changes in microstructure after roasting

**FIGURE 1:**
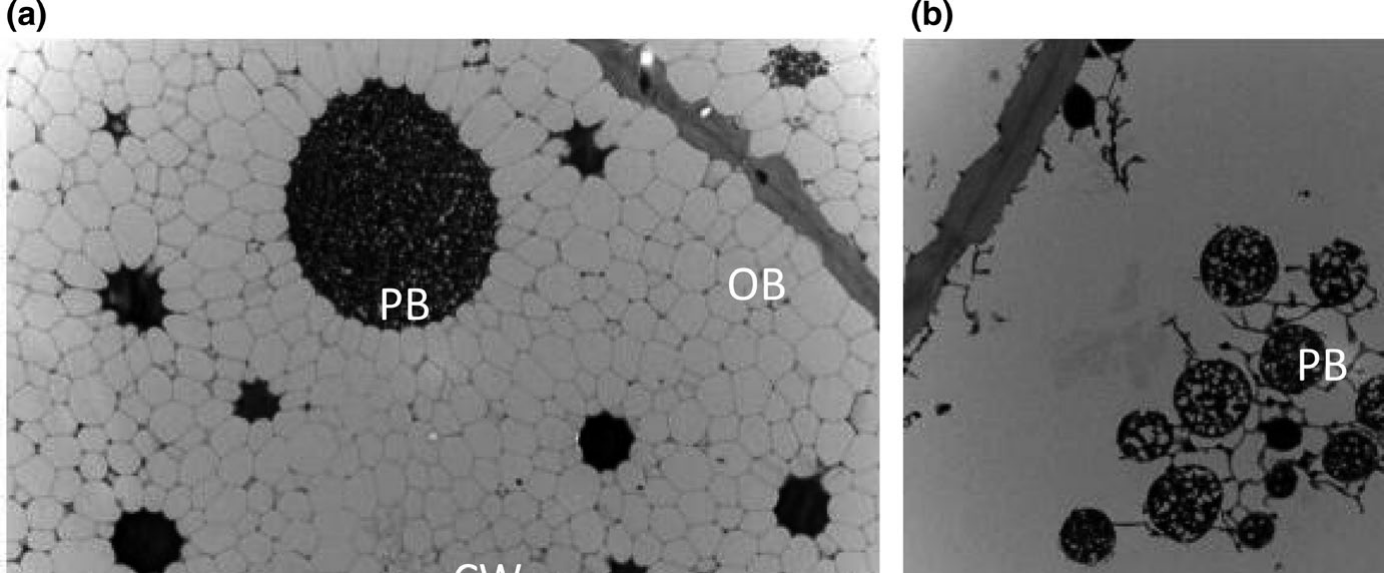
TEM images of raw (a) and roasted (b) macadamia nuts (5,000×)

Transmission electron microscopy imaging of raw nuts (Figure 1a) showed a regular structure with spherical protein bodies (PB), surrounded by much smaller, well‐defined, spherical oil bodies (OB). After roasting, the membrane structure of the oil bodies was disrupted, resulting in an apparently continuous oil phase, the cell wall (CW) boundary became fuzzy and irregular and the oil bodies, and protein bodies coalesced. The images clearly demonstrate that roasted treatment disrupts the membranes of oil bodies as well as other intracellular organelles, which makes the oil spill more easily (Niu et al., 2015). Roasting resulted in an improvement in lipid extractability; the crude fat content of both varieties apparently increased by 2% (Table 1). Incorporation of roasting in standard oil extraction methods markedly increased lipid extraction efficiency (Yoshida et al., 2006).

### 3.2 Physicochemical properties of raw and roasted nuts

The effects of roasting on the composition of macadamia nuts were determined (Table 2). The moisture content of both varieties decreased from ~1.5 to 1%, the water activity of Guire 1 decreased from 0.62 to 0.38, and that of O.C decreased from 0.60 to 0.36. Water activity has a great influence on product storage stability and may cause changes in texture, color, flavor, aroma, stability, and acceptability, of both processed and raw nuts. The optimum water activity for storage of macadamia nuts is less than 0.53 at 25°C (Pankaew et al., 2016). Roasting of macadamia nuts, therefore, reduces their water activity to within the optimum range for storage stability.

**TABLE 2:** Physicochemical properties and nutrition components of raw and roasted macadamia nuts

Physicochemical properties	Guire 1		Own choice	
	Raw	Roasted	Raw	Roasted
Moisture (%)	1.46 ± 0.12^a^	1.03± 0.06^b^	1.41 ± 0.09^a^	0.98 ± 0.04^c^
Water activity	0.62 ± 0.01^a^	0.38 ± 0.01^b^	0.60 ± 0.00^a^	0.36 ± 0.00^b^
Crude fat (%)	74.10 ± 2.11^c^	76.30 ± 1.85^b^	75.42 ± 0.63^bc^	77.39 ± 0.54^a^
Crude protein (%)	8.81± 1.25^a^	8.63 ± 0.37^b^	8.78 ± 1.82^a^	8.61 ± 0.21^b^
Ash (%)	2.20 ± 0.35^c^	2.70 ± 0.24^a^	2.40 ± 0.26^b^	2.31 ± 0.22^bc^
Crude fiber (%)	4.10 ± 1.71^a^	3.87 ± 0.21^b^	3.60 ± 0.81^c^	3.51 ± 0.30^c^
Carbohydrate (%)	2.26 ± 0.37^c^	2.15 ± 0.51^c^	3.13 ± 0.58^a^	2.47 ± 0.22^b^
Peroxide value (meq/kg, PV)	0.34 ± 0.01^bc^	0.49 ± 0.02^b^	0.19 ± 0.01^c^	0.63 ± 0.01^a^
Acid value (mg KOH/g, AV)	0.76 ± 0.01^c^	0.89 ± 0.07^b^	0.64 ± 0.06^c^	0.81 ± 0.06^a^
OSI (h)	17.56 ± 0.23^b^	21.37 ± 0.35^a^	16.39 ± 0.19^b^	20.85 ± 0.45^a^

Note

Different superscript letters indicate that the means were significantly different (*p* < .05).

The acid value (AV) and peroxide value (POV) have been adopted as primary indicators of hydrolytic rancidity and hydroperoxide formation in tree nuts, during the initial phases of lipid oxidation (Sinanoglou et al., 2014; Wang, Zhang, Johnson, et al., 2014). In addition, they are important indicators of shelf life. There was a significant increase in the peroxide and acid values of both varieties after roasting (Table 2). However, the after‐roasting values were still well below the upper limits (PV ≤ 3 meq/kg, AV ≤ 3 mg KOH/g) of the Chinese National Standard (GB 19300‐2014) and the UNECE Standard DDP‐23, Macadamia Kernels[S] (2011). In addition, the oxidative stability index (OSI) of the roasted nuts was greatly improved. This results from the Maillard browning reaction between sugars and amino acids (Chang et al., 2016), which can improve antioxidant capacity (Ng et al., 2014).

### 3.3 Nutritional components in raw and roasted nuts

Roasting moderately decreased the measured crude protein, crude fiber, and carbohydrate contents of Guire 1 and O.C (Table 2). In particular, roasting does not change the content of anything, apart from moisture, but it does change the measurable, or available content, by changing extractability. From the results in Table 2, there was significant change in the other nutritional components after roasting. The increase in available oil content indicated that the fat component of the nut kernel flour was largely removed during protein bodies coalesced. However, the roasted nuts were lower in moisture, crude fiber, ash, and crude protein, but higher in carbohydrate when compared to the respective dried raw sample (Table 2).

The available sterol content increased by 13.1% in Guire 1 and 20.6% in O.C after roasting (*p* < .05) (Table 3), and the total available phenolic contents increased significantly (*p* < .05), by 25.6% in Guire 1 and 19.4% in O.C. Roasting significantly increased the available levels of sterols and polyphenols, in agreement with previous reports, and appears to result from increased extractability of the isoforms because of heat treatment. These changes are accompanied by migration of some lipid species within the cell and improved bioavailability of some minor components (Moghaddam et al., 2016; Sinanoglou et al., 2014). The thiamine content in macadamia nuts was the highest reported for tree nuts, although roasting resulted in a moderate decrease in thiamine content in Guire 1, by 16.2% and in O.C by 17.8%, consistent with an earlier report (Stuetz et al., 2017).

**TABLE 3:** Lipophilic compounds in raw and roasted macadamia nuts

Chemical composition	Guire 1		Own Choice	
	Raw	Roasted	Raw	Roasted
Sterol (mg/100 g)	107.16 ± 2.36^c^	121.19 ± 1.32^b^	115.58 ± 2.64^b^	139.38 ± 2.18^a^
Tocopherol (mg/100 g)	0.27 ± 0.05^a^	0.11 ± 0.02^b^	ND	ND
Tocotrienol (mg/100 g)	5.96 ± 0.19^b^	5.83 ± 0.16^c^	6.26 ± 0.14^a^	6.11 ± 0.21^b^
Squalene (mg/100 g)	8.23 ± 0.13^c^	10.17 ± 0.21^a^	7.96 ± 0.16^c^	9.86 ± 0.18^b^
Total phenolic content (mg GAE/100 g)	60.52 ± 0.95^c^	75.96 ± 1.03^b^	68.32 ± 1.12^c^	81.56 ± 0.86^a^
Thiamine (mg/100 g)	0.37 ± 0.06^a^	0.31 ± 0.02^b^	0.28 ± 0.04^c^	0.23 ± 0.01^d^

Note

Total phenolics in nuts were expressed as gallic acid equivalents (GAE); ND, not detected.

Roasting of nuts promotes cellular swelling and rupture; for example, considerable swelling of the cell wall and middle lamella of roasted almonds resulted in improved release of lipids from intact cell walls during digestion, indicating that diffusion of enzymes, bile salts, and lipid products into nut material in the GI tract may be facilitated by roasting (Bolling et al., 2010). The yield of soluble phenolic extracts from macadamia nut kernels significantly increased after roasting; the same result was found in cashew nuts (Chandrasekara & Shahidi, 2011).

### 3.4 Analysis of lipid composition in raw and roasted nuts

Gas chromatography (GC) analysis identified 11 FAs in macadamia nuts (Table 4) and the FA compositions of raw and roasted nuts were consistent with a previous report (Sinanoglou et al., 2014). The fatty acid profile mainly consisted of monounsaturated fatty acids (MUFA), predominantly oleic acid (C18:1 n‐9) and palmitoleic acid (C16:1 n‐7) acids, along with substantial proportions of the saturated palmitic acid (C16:0) and stearic acid (C18:0). In general, fatty acid percentages decreased in the order of monounsaturated > saturated > polyunsaturated (MUFA > SFA > PUFA), consistent with a previous report (Xu et al., 2018). There was no difference in FA composition between raw and roasted nuts, but that did not necessarily mean there were no differences in the TAG or FFA profiles. Thus, further analysis was performed.

**TABLE 4:** Fatty acid composition of raw and roasted macadamia nuts (relative content, %, w/w)

FAs	Guire 1		Own choice	
	Raw	Roasted	Raw	Roasted
Myristic acid (M, C14:0)	0.34 ± 0.01^b^	0.39 ± 0.00^b^	0.57 ± 0.03^a^	0.61 ± 0.01^a^
Palmitic acid (P, C16:0)	8.86 ± 0.05^a^	9.01 ± 0.06^a^	8.16 ± 0.07^b^	8.25 ± 0.09^b^
Palmitoleic acid (Po, C16:1)	18.73 ± 0.13^b^	18.53 ± 0.16^b^	19.76 ± 0.15^a^	19.65 ± 0.11^a^
Stearic acid (S, C18:0)	3.13 ± 0.08^d^	3.35 ± 0.13^c^	3.43 ± 0.05^b^	3.68 ± 0.11^a^
Oleic acid (O, C18:1)	61.18 ± 0.21^a^	60.96 ± 0.14^a^	59.65 ± 0.31^b^	59.31 ± 0.16^b^
Linoleic acid (L, C18:2)	2.26 ± 0.04^b^	1.92 ± 0.02^c^	2.87 ± 0.07^a^	2.76 ± 0.04^a^
Arachidic acid (A, C20:0)	2.25 ± 0.01^c^	2.56 ± 0.07^b^	2.93 ± 0.03^a^	3.06 ± 0.08^a^
Gondoic acid (G, C20:1)	2.17 ± 0.09^a^	2.05 ± 0.02^a^	2.02 ± 0.04^b^	2.01 ± 0.05^b^
Behenic acid (B, C22:0)	0.73 ± 0.04^b^	0.86 ± 0.02^b^	0.61 ± 0.06^a^	0.67 ± 0.03^a^
Tetracosanoic acid (T, C24:0)	0.35 ± 0.02^a^	0.37 ± 0.06^a^	ND	ND
Saturated fatty acids (SFAs)	15.66 ± 0.15^b^	16.54 ± 0.12^a^	15.70 ± 0.19^b^	16.27 ± 0.11^a^
Unsaturated fatty acids (USFAs)	84.34 ± 0.17^a^	83.46 ± 0.11^b^	84.30 ± 0.16^a^	83.73 ± 0.08^b^

Note

Values are the mean of three determinations from two independent experiments (*n* = 3). ND, Not detected.

**FIGURE 2:**
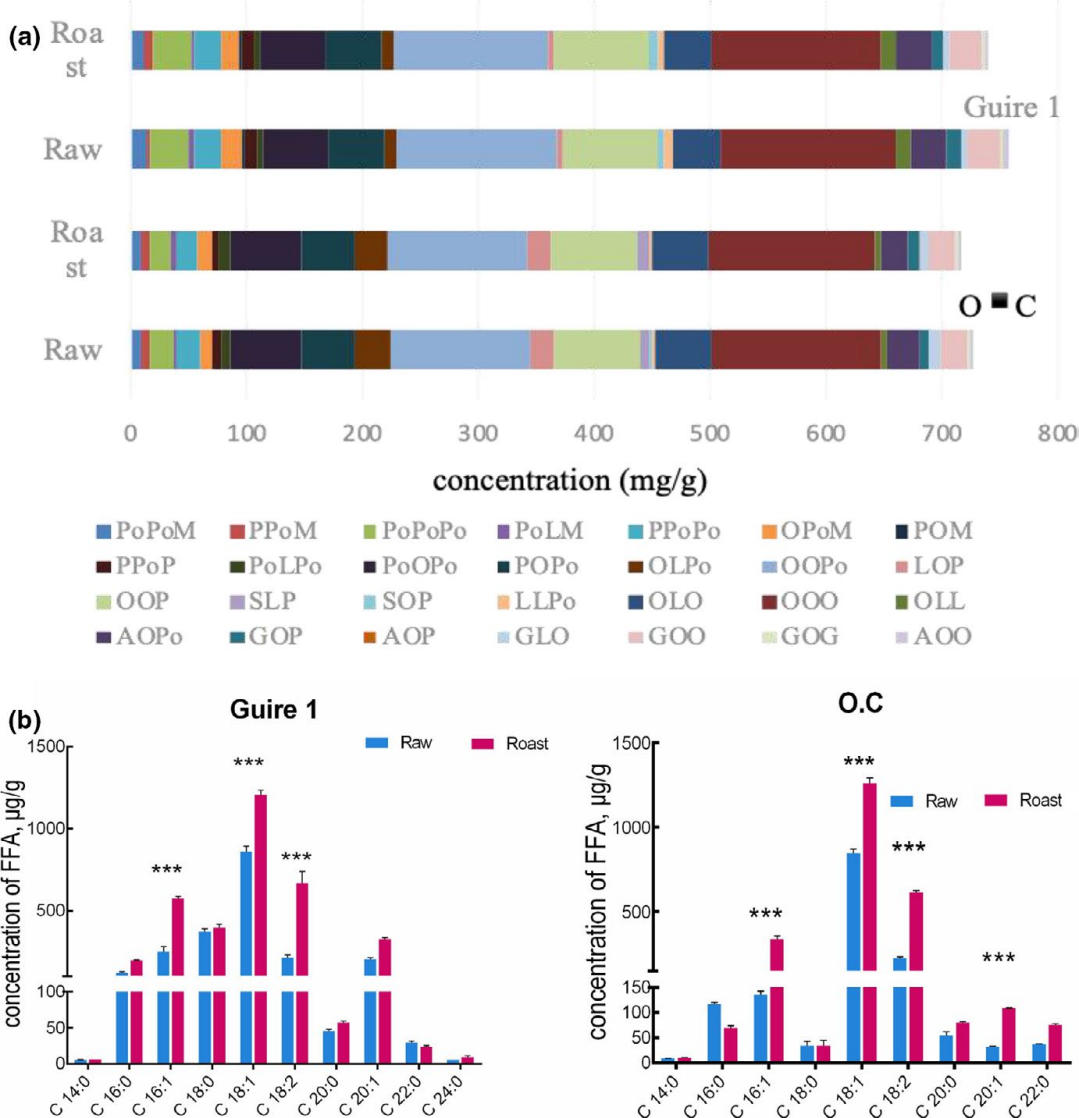
TAG composition (mg/g) (a) and FFA concentrations (μg/g) (b) in raw and roasted macadamia nuts; the lines in the graph (a) represent the concentration of each triglyceride molecule (mg/g); the original data of Figure 2 have been provided in the supplemental materials as Table S1‐S2

The shotgun lipidomics approach identified 38 lipid molecular species in macadamia nuts, which were characterized and quantified, including 28 TAGs and 10 FFAs (Figure 2a). The two varieties of macadamia nuts contained the same amounts of triglycerides, and the fatty acid composition in the TAGs was consistent with the GC analysis results except for C24:0. This may result from greater sensitivity of MS, compared with GC, so the former can detect some trace fatty acids. The most abundant TAG species of raw dried and roasted macadamia nuts were OOO (1,2,3‐trioleic acid‐triglyceride), OOP_O_ (1,2‐oleic acid, 3‐palmitoleic acid‐triglyceride), OOP (1,2‐oleic acid, 3‐palmitate‐triglyceride), and P_O_OP_O_ (1,3‐ palmitoleic acid, 2‐oleic acid‐ triglyceride), the contents >50 mg/g, which is consistent with previous reports (Sinanoglou et al., 2014). The total TAG content of the two varieties decreased slightly after roasting, but there was no significant difference. TAG is the main component of macadamia nut oil, accounting for 97.5% and has a great impact on the function and nutritional characteristics of the nut oil. In summary, roasting had a negligible effect on TAG content or composition.

Ten FFAs were quantified, the most abundant being oleic acid (C18:1), followed by linoleic acid (C18:2), then palmitoleic acid (C16:1) (Figure 2b). Very small amounts of myristic acid (C14:0) and tetracosanoic acid (C24:0) were also detected. The contents of most FFAs increased after roasting and the contents of palmitoleic acid (C16:1), oleic acid (C18:1), and linoleic acid (C18:2) increased significantly, for example, the C16:1 in Guire 1 from 280.95 to 577.95 μg/g, the C18:1 in O.C from 819.14 to 1,308.87 μg/g, the C18:1 in O.C from 819.14 to 1,308.87 μg/g, the C18:1 in Guire 1 from 838.36 to 1,220.25 μg/g, the C18:2 in O.C from 210.79 to 601.67 μg/g, resulting from hydrolysis of TAGs into FFAs by lipase, or nonenzymic hydrolysis. FFAs liberated from TAGs were related to the fatty acid composition and positional distribution (Liu et al., 2018). Fatty acids in the 2‐position of TAG molecules are more easily hydrolyzed to form FFAs (Lu et al., 2019; Xie et al., 2019). Generally, saturated FAs, especially C18:0, mostly occupied the 1 and 3 positions, whereas unsaturated FAs, particularly C18:1 and C18:2, were primarily concentrated in 2‐position of TAGs (Xie et al., 2019). This explains the larger increases in the FFAs C18:1, C18:2, and C16:1. Since the total TAG content is more than 95.5% and the total FFA content is less than 0.15%. Therefore, even a small amount of TAG hydrolysis can cause a significant increase in FFA content during roasting (Sinanoglou et al., 2014).

Lipid oxidation was used to evaluate postharvest quality of nuts during processing and storage, FFAs are more susceptible to oxidation than triglycerides (TAGs), and an FFA concentration exceeding 1% decreased the shelf life of hazelnuts with previous reports (Moscetti et al., 2012). Therefore, the FFA levels are a more reliable quality indicator of macadamia kernels (Toth, 2017). From Figure 2, Our results suggest that the production of the total FFAs from Guire 1 and O.C kernels was not accelerated significantly by high temperature, although the content of several unsaturated free fatty acids has increased. The low production of free fatty acids, despite the exposure of kernels to high temperature, was possibly because there was insufficient moisture in the kernels for lipid hydrolysis (Walton & Wallace, 2011). The Australian Macadamia Society (AMS) considers 0.5% FFA acceptable of kernel quality standard for processors. Combined with the results of Table 1, the quality and shelf life of macadamia nuts did not decrease after roasting.

### 3.5 Sensory evaluation

**FIGURE 3:**
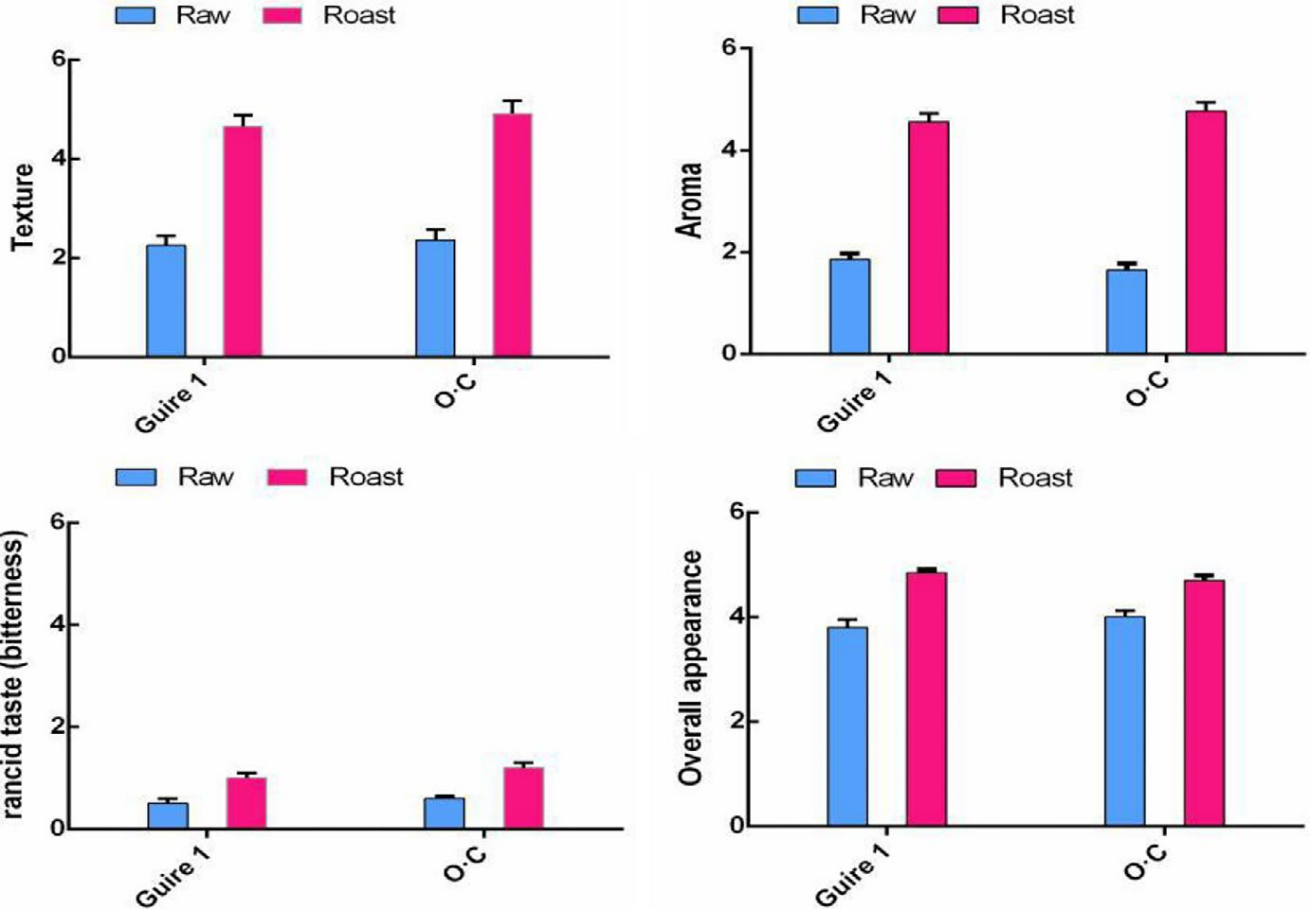
Sensory evaluation results of raw and roasted macadamia nuts

The sensory evaluation of raw and roasted macadamia nuts showed that the texture, aroma, and overall appearance of both macadamia cultivars had been significantly (*p* < .05) improved by the roasting process (Figure 3). All members of sensory evaluation team expressed that both varieties of roasted kernels were crisp, delicate, and uniform in good color and aroma, whereas the dried raw kernels had a smooth texture and almost no aroma.

In particular, the taste and aroma scores more than doubled, whereas the increase in rancid taste (bitterness) was not noticeable, because the increased scores were still below 1, indicating that roasting did not produce detectable off‐flavors, in agreement with previous reports (Walton et al., 2013). Moisture loss is considered to be the main factor in texture development of roasted kernels, leading to increased crispiness (Schlormann et al., 2015). Kernels become crisper after roasting and are preferred by consumers (Liaotrakoon et al., 2016). The roasting process is responsible for the development of the typical taste and the crunchy texture of macadamia nuts, resulting from structural and chemical changes, such as lipid oxidation and Maillard reactions (Fischer et al., 2017; Liu et al., 2018). The enhancement of aroma in roasted nuts can be attributed to the generation of aromatic compounds such as benzaldehyde, methylphenol, alcohol, and alkylbenzenes during roasting process (Fischer et al., 2017). Maillard browning reaction and caramelization also promote the generation of flavor compounds (Poogungploy et al., 2018). According to the sensory evaluation results, under the recommended roasting conditions, macadamia nuts undergo very little browning, have improved sensory quality, and are acceptable to consumers.

## 4 CONCLUSION

In this study, changes in sensory quality, nutritional composition, and lipid oxidation were examined in raw and roasted macadamia nuts, showing that the roasting process significantly improves the kernel quality of “Guire 1” and “O.C” macadamia nuts. The low PV value, high concentration of total polyphenols, and good sensory quality indicated that lipid oxidation in the roasting process is acceptably low. Changes of the chemical composition during roasting, low levels of lipid oxidation, and hydrolysis generate new flavor and aroma compounds that produce the characteristic roast nut flavor. From the above research results, both raw and roasted macadamia nuts have high nutritional value.

## CONFLICT OF INTEREST

The authors have declared no conflict of interest.

## Supporting information

Table S1‐S2

## Data Availability

Research data are not shared.
